# A Systematic Review of the Effects of Temperature on *Anopheles* Mosquito Development and Survival: Implications for Malaria Control in a Future Warmer Climate

**DOI:** 10.3390/ijerph18147255

**Published:** 2021-07-07

**Authors:** Thomas P. Agyekum, Paul K. Botwe, John Arko-Mensah, Ibrahim Issah, Augustine A. Acquah, Jonathan N. Hogarh, Duah Dwomoh, Thomas G. Robins, Julius N. Fobil

**Affiliations:** 1 Department of Biological, Environmental and Occupational Health Sciences, School of Public Health, University of Ghana, Accra 00233, Ghana; pkbotwe@ug.edu.gh (P.K.B.); jarko-mensah@ug.edu.gh (J.A.-M.); ibrahimissah111@gmail.com (I.I.); aaacquah@st.ug.edu.gh (A.A.A.); jfobil@ug.edu.gh (J.N.F.); 2 Department of Environmental Science, Kwame Nkrumah University of Science and Technology, Kumasi 00233, Ghana; jhogarh@gmail.com; 3 Department of Biostatistics, School of Public Health, College of Health Sciences, University of Ghana, Accra 00233, Ghana; duahdwomoh@gmail.com; 4 Department of Environmental Health Sciences, University of Michigan, 1415 Washington Heights, Ann Arbor, MI 48109, USA; trobins@umich.edu

**Keywords:** *Anopheles* mosquito, body size, fecundity, gonotrophic cycle, immature stage, insecticide, longevity, temperature

## Abstract

The rearing temperature of the immature stages can have a significant impact on the life-history traits and the ability of adult mosquitoes to transmit diseases. This review assessed published evidence of the effects of temperature on the immature stages, life-history traits, insecticide susceptibility, and expression of enzymes in the adult *Anopheles* mosquito. Original articles published through 31 March 2021 were systematically retrieved from Scopus, Google Scholar, Science Direct, PubMed, ProQuest, and Web of Science databases. After applying eligibility criteria, 29 studies were included. The review revealed that immature stages of *An. arabiensis* were more tolerant (in terms of survival) to a higher temperature than *An. funestus* and *An. quadriannulatus*. Higher temperatures resulted in smaller larval sizes and decreased hatching and pupation time. The development rate and survival of *An. stephensi* was significantly reduced at a higher temperature than a lower temperature. Increasing temperatures decreased the longevity, body size, length of the gonotrophic cycle, and fecundity of *Anopheles* mosquitoes. Higher rearing temperatures increased pyrethroid resistance in adults of the *An. arabiensis* SENN DDT strain, and increased pyrethroid tolerance in the *An. arabiensis* SENN strain. Increasing temperature also significantly increased Nitric Oxide Synthase (NOS) expression and decreased insecticide toxicity. Both extreme low and high temperatures affect *Anopheles* mosquito development and survival. Climate change could have diverse effects on *Anopheles* mosquitoes. The sensitivities of *Anopeheles* mosquitoes to temperature differ from species to species, even among the same complex. Notwithstanding, there seem to be limited studies on the effects of temperature on adult life-history traits of *Anopheles* mosquitoes, and more studies are needed to clarify this relationship.

## 1. Introduction

Climate change is one of the most significant global challenges in the twenty-first century [1]. It is a global phenomenon [2,3,4], largely caused by anthropogenic activities, and poses significant risks to a broad range of human and natural systems [5]. Climate change is being experienced through an increase in global temperatures, sea-level rise, shrinking ice sheets, warming oceans, Arctic sea ice decline, glacial retreat, increasing extreme events, ocean acidification, and decreased snow cover [6]. Climate change may affect human health in many ways, including affecting livelihood and food security [7,8]. In addition, climate change could directly influence the patterns of infectious diseases and vector-borne diseases [9] and modify vector distribution and the extension of geographical ranges of mosquitoes such as the malaria vector [10]. However, there is a narrow understanding of how climatic factors such as temperature affect the development and survival of *Anopheles* mosquitoes, which are the primary vectors of human malaria.

*Anopheles* mosquitoes are poikilotherms with life history characteristics strongly dependent on the ambient temperature. These characteristics include the length of the gonotrophic cycle, fecundity, biting rate, longevity, and development of immature mosquitoes [11]. Thus, any factor that alters these characteristics can potentially affect the ability of mosquitoes to transmit diseases. Climate parameters such as temperature, humidity, and rainfall noticeably influence both the mosquito’s life-history traits and the parasite’s sporogonic development within their bodies [12,13,14]. Temperature also affects the mosquito’s immune system [15,16,17]. Moreover, most of the interventions aimed at controlling *Anopheles* mosquito populations generally depend on insecticides. The efficacy of these insecticides is dependent not only on the active ingredient but also on other factors, such as ambient temperature [18,19,20].

Most studies that have considered the effects of temperature on mosquito development and survival have focused more on species such as *Culex* and *Aedes* [11,21,22,23]. For instance, Ezeakacha and Yee [21] investigated the role of temperature in affecting the carry-over effects and larval competition in *Aedes albopictus* mosquitoes and found that temperature affected both the immature and adult mosquitoes. The conditions at the immature stages of mosquitoes influence the quality of adult life [24] as well as the determination of the age structure of the adult population [25]. In addition, studies on *Anopheles* mosquitoes have considered the effects of temperature on different aspects of the life-history traits [26,27,28,29]. No study has attempted to synthesize all the studies on the different species of *Anopheles* mosquitoes into a single study to determine the effects of temperature on *Anopheles* mosquitoes. In this systematic review, we assembled and evaluated the available evidence showing the relationship between temperature and the immature stages, life-history traits of adults, insecticide susceptibility, and enzyme expression or immune responses in the adult *Anopheles* mosquito.

## 2. Methods

This systematic review’s findings were reported following the Preferred Reporting Items for Systematic Reviews and Meta-Analyses (PRISMA) guidelines [30]. This systematic review has been registered with PROSPERO (https://www.crd.york.ac.uk/prospero/display_record.php?ID=CRD42020196407 (accessed on 24 June 2021)) and had the registration number CRD42020196407 assigned to it.

### 2.1. Eligibility Criteria

To assess the effects of temperature on *Anopheles* mosquito development and survival, original studies that considered either the immature or adult *Anopheles* mosquitoes irrespective of the complex were included. In addition, this review included studies that considered either field studies, laboratory studies, or both. Studies that evaluated any of the following outcomes; development rate, longevity, fecundity, length of the gonotrophic cycle, biting rate, susceptibility to insecticides, and the expression of enzymes and genes were also included. However, studies that did not focus on *Anopheles* mosquitoes and any of the listed outcomes were excluded. Studies not written in English were also excluded. In addition, review papers, books, opinions, scientific reports and perspectives, and duplicate records were all excluded.

### 2.2. Search Strategy and Selection Criteria

An initial search was conducted to identify keywords and synonyms. Research articles published up to March 2021 were systematically retrieved from PubMed, Science Direct, Scopus, ProQuest, Web of Science, and Google Scholar databases. This search was conducted in September 2020 and updated in March 2021 to retrieve any current articles. A detailed search strategy (Table S1) was developed and used in the article searching stage of this systematic review. The search strategies used terms such as *Anopheles* mosquito, malaria, temperature, temp*, season*, survival, longevity, etc. Combinations of different search strings and search terms were employed for each electronic database to enhance the search’s sensitivity and specificity. Articles were exported into EndNote reference manager (version X9). Three independent reviewers (T.P.A., A.A.A., and I.I.) screened the search results’ title and abstract to assess potentially eligible studies. Full-text articles were then retrieved and reviewed to obtain the final set of articles included in the review. Disagreements in the screening and selection of articles were resolved by dialogue, and a consensus was reached at all stages.

### 2.3. Data Extraction

A data-extraction form was pretested by one reviewer (T.P.A.). The form was later revised to include author details, study type, study location, *Anopheles* species considered, the rearing conditions, and the outcome of interest. Data from the included studies were first extracted and reviewed by three authors (T.P.A., I.I., and A.A.A.) independently and later jointly to resolve disagreements. Where necessary, corresponding authors of some of the included studies were contacted for further information.

### 2.4. Risk of Bias Assessment

Three authors (T.P.A., A.A.A., and I.I.) independently performed the included studies’ risk of bias. Disagreements were resolved through discussion and involvement of a fourth person where necessary. The risk of bias was assessed using the Systematic Review Center for Laboratory Animal Experimentation’s (SYRCLE’s) tool for animal studies [31]. The tool comprises ten (10) domains with six (6) types of bias: selection bias, performance bias, detection bias, attrition bias, reporting bias, and others. The ten (10) items are structured in sub-sections in question forms that require a “Yes (low risk),” “No (high risk),” or “Unclear (unclear risk)” answer.

### 2.5. Data Analysis

A narrative synthesis of all the included studies was performed based on the outcome of interest, and the findings were reported in tabular form for easy interpretation and understanding. All the included studies were quantitative; however, this review did not include a meta-analysis.

## 3. Results

### 3.1. Search Results

**Figure 1:**
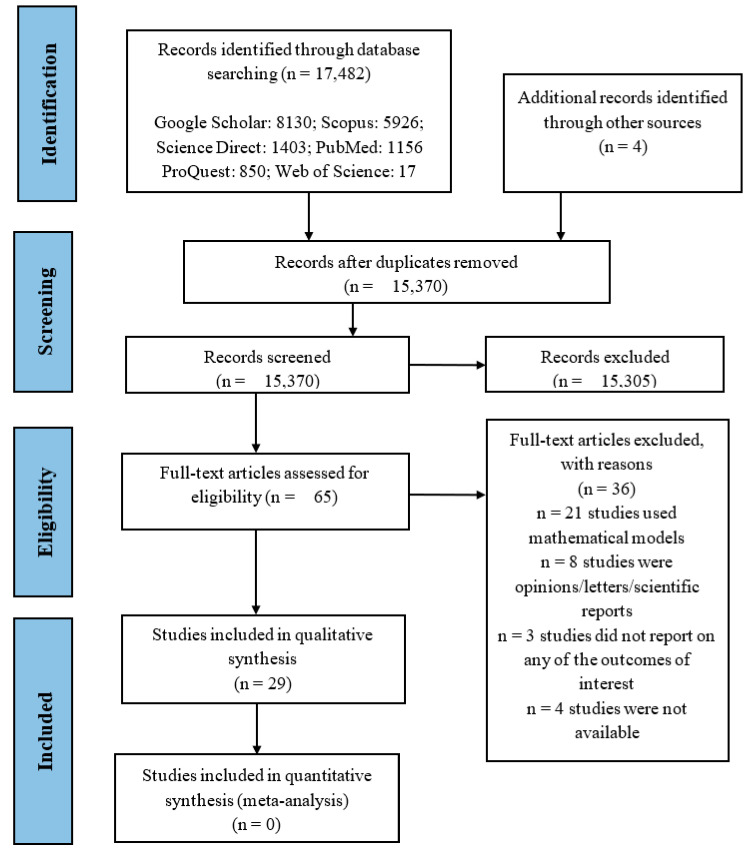
PRISMA flow diagram of search phases with numbers of studies included/excluded at each stage.

From the search, 8130, 5926, 1403, 1156, 850, and 17 records were retrieved from Google Scholar, Scopus, Science Direct, PubMed, ProQuest, and Web of Science databases, respectively (Table S1). Four (4) additional articles were obtained through contacts with experts in the field and screening the reference lists of included studies. After removing duplicates and screening titles and abstracts, 65 records were included for full-text assessment. Thirty-six (36) articles were excluded with reasons (Table S2), while 29 articles [15,16,18,19,26,27,28,29,32,33,34,35,36,37,38,39,40,41,42,43,44,45,46,47,48,49,50,51,52] fully met the inclusion criteria (Figure 1).

### 3.2. Study Characteristics

**Figure 2:**
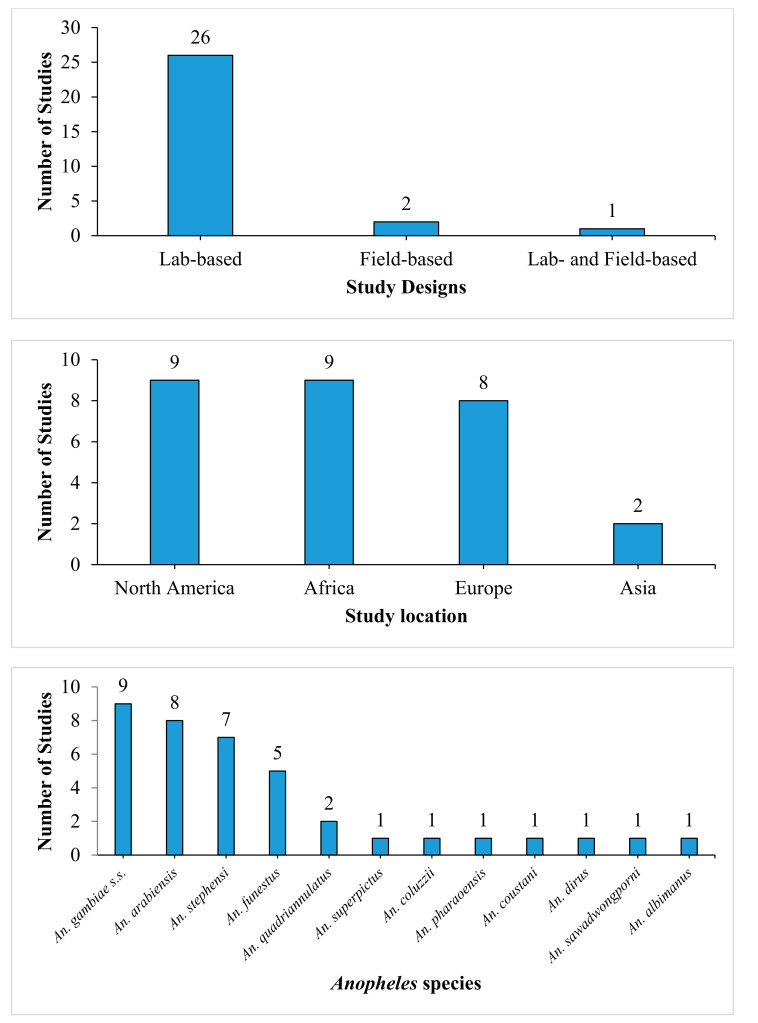
Characteristics of included studies.

The included studies consisted of twenty-six (26) laboratory-based studies, two (2) field-based studies, and one (1) study that employed both study designs. Different species of *Anopheles* mosquitoes were reported in the included studies. Most of the included studies were conducted in Africa (9), North America (9), Europe (8), and Asia (2). One study did not indicate the study location. About 12 different *Anopheles* species were reported in the 29 studies, and the majority of these species were *An. gambiae* s.s. (9), *An. arabiensis* (8), *An. stephensi* (7), and *An. funestus* (5) (Figure 2).

### 3.3. Risk of Bias Assessment

#### 3.3.1. Selection Bias

Except for one study [43], which was at low risk, all 28 studies reviewed were at high risk of sequence generation. With baseline characteristics, only two studies [37,46] had unclear risk. Additionally, the remaining 27 studies had low risk. Concerning allocation concealment, the risk was unclear in twelve (12) studies [26,27,32,33,34,35,36,37,38,39,40,41], while the remaining fifteen (17) studies were at high risk. However, the absence of sequence generation and allocation concealment is unlikely to influence the findings (Table 1).

#### 3.3.2. Blinding (Performance and Detection Bias)

Unlike drug trials, where it is easy to blind investigators from the intervention being administered, the investigator is not usually blinded to the treatments in most insect studies. Blinding does not apply to this systematic review.

#### 3.3.3. Randomization (Performance and Detection Bias)

This bias does not apply to this systematic review.

#### 3.3.4. Bias (Attrition and Reporting)

All the 29 studies had a low risk of attrition and reporting bias. The studies presented a detailed and consistent reporting of all outcomes prespecified in the methods section (Table 1).

#### 3.3.5. Other Sources of Bias (Funding Source and Rearing of Mosquitoes)

Except for eight (8) studies [32,33,37,41,46,47,49,51] that failed to disclose funding sources, the majority of the studies (20) declared the source of funding and funders did not influence the results. However, one study [50] had an unclear risk. Although the study indicated that funding was acquired, it did not state or provide enough information to judge funding sources.

In assessing how temperature affects *Anopheles* mosquitoes, most of the studies reared the mosquitoes in incubators from either the egg or larval stage to adult. Rearing mosquitoes in incubators from the egg or larval to the adult stages may better assess the effect of temperature on the mosquito. Nine (9) studies [15,16,18,19,28,37,43,45,46] were at high risk of bias based on mosquito rearing conditions (Table 1). In some of these studies, adult mosquitoes were only exposed to the selected temperature regimes before outcome assessment, which may affect the study’s outcome.

### 3.4. Effects of Temperature on the Immature stages of Anopheles Mosquitoes

Sixteen (16) studies assessed the effects of temperature on different *Anopheles* species (Table 2). These studies considered larval and pupal development and survival, as well as egg hatchability. The way temperature affected the immature stages of mosquitoes differed from species to species, even among the same complex. The immature stages of *An. arabiensis* were more tolerant (in terms of survival) to a higher temperature than *An. funestus* [28], and *An. quadriannulatus* [38]. In addition, *Anopheles arabiensis* showed faster development rates (in days) compared to *An. funestus* [42] and *An. quadriannulatus* [38].

The minimum and maximum temperatures from these studies were 10 and 40 °C, respectively. One study [27] indicated that higher temperatures (23 to 31 °C) resulted in smaller larval sizes and slowed the development from hatching to adult emergence. However, most studies [29,32,35,41,48,49] observed that increasing temperature reduced the development time (in days) of the immature stages. For instance, Phasomkusolsil et al. [49] observed that *An. dirus* and *An. sawadwongporni* larvae reared at 30 °C displayed a significantly shorter developmental time (approximately 7–8 days) than those reared at 23 °C (12–14 days) (*p* < 0.05). Higher temperatures (30 and 35 °C) significantly increased larval development rates in two *An. arabiensis* strains–SENN DDT (one-way ANOVA: *p* < 0.01; F = 15.1) and SENN (one-way ANOVA: *p* < 0.01; F = 12.4) relative to their respective 25 °C control cohorts [29].

An increase in temperature significantly decreased the time to pupation of *An. gambiae* s.s. larvae from 9.2 ± 0.05 days at 21 °C to 8.3 ± 0.04 days at 25 °C and 7.8 ± 0.05 days at 29 °C [34], and increased larval mortality [26,36]. Christiansen-Jucht et al. [26] reported that, an increase in temperature at varying intervals of 4 °C (from 23 °C to 27 °C, *p* < 0.001), 8 °C (from 27 °C to 35 °C, *p* < 0.001), and 12 °C (from 23 °C to 35 °C, *p* < 0.001) significantly decreased larval survival.

Increasing temperature decreased the time to hatching but not the hatching rate of *Anopheles* eggs. For instance, hatching of *An. arabiensis* eggs were fastest at 27 °C and slowest at 22 °C; nevertheless, most of the eggs hatched within two days irrespective of the water temperature [44]. There was no significant difference (*p* > 0.05) between the mean hatching rate of *An. dirus* and *An. sawadwongporni* eggs reared at 23 °C and 30 °C [49]. However, extremely high temperatures can affect the hatchability of eggs. Impoinvil et al. [40] observed that incubating eggs at 42 °C for a day resulted in a low mean hatching count relative to the other temperatures. There was no hatching of eggs when the incubation period was extended to 3, 7, and 10 days.

### 3.5. Effects of Temperature on the Life History Traits of Adult Mosquitoes

#### 3.5.1. Longevity

Five (5) studies [29,32,34,39,46] assessed the longevity of different *Anopheles* mosquitoes from either field or laboratory populations (Table 3). Olayemi et al. [46] reported that the longevity and survival rate of *An. gambiae* mosquitoes were higher in the rainy season (17.48 ± 2.92 days and 84.5% ± 10.46%, respectively) than in the dry season (7.29 ± 2.82 days and 57.47% ± 14.9%, respectively). The rainy season is associated with cooler temperatures and the dry season with hotter temperatures. In addition, Faiman et al. [39] observed that the longevity of *An. coluzzii* increased at a lower temperature; however, the main effect of temperature was not statistically significant (*p* = 0.072). They detected higher longevity at a lower temperature in each experiment and between 22 °C and 23.5 °C (*p* < 0.001) but not between experiments at 27 °C (*p* = 0.072). Similar trends were reported by Aytekin et al. [32] and Barreaux et al. [34]. More adult *An. gambiae* s.s. died with every increase in temperature compared to the baseline temperature (i.e., 23 °C). All the *p*-values were statistically significant (*p* < 0.001) for comparisons of 27 °C vs. 23 °C, 31 °C vs. 27 °C, and 31 °C vs. 23 °C [26].

#### 3.5.2. Body Size

In most mosquito studies, wing length has been used as a proxy to measure mosquito body size. All the seven (7) studies [27,32,33,34,37,41,49] reported on body size showed a decrease in wing length and body weight with increasing temperature (Table 4). For instance, *An. dirus* and *An. sawadwongporni* mosquitoes reared at 23 °C were significantly heavier and longer than those reared at 30 °C (*p* < 0.05) [49]. Barreaux et al. [34] also observed that the wing length of *An. gambiae* s.s. mosquitoes decreased significantly (F(2, 181) = 35.7, *p* < 0.0001) with increasing temperature from 3.27 mm at 21 °C to 3.23 mm at 25 °C and 3.02 mm at 29 °C. Expect for Charlwood and Bragança [37], who measured body sizes of field-collected mosquitoes; all the remaining studies measured the body size of adult mosquitoes reared from the egg stage through to adult. Only Christiansen-Jucht et al. [27] measured the size of the larvae in addition to the adult mosquitoes.

#### 3.5.3. Fecundity, Length of the Gonotrophic Cycle, and Biting Rate

Four (4) studies [27,32,43,49] assessed the effects of temperature on fecundity. Similarly, four studies [43,47,50,51] also assessed the effects of temperature on gonotrophic cycle length, with only one study [50] considering biting rate (Table 5). Three of the studies reported on fecundity [27,32,49] showed a decrease in fecundity with increasing temperature. For example, the mean number of eggs laid by *An. dirus* and *An. sawadwongporni* mosquitoes reared at 23 °C was significantly higher than those reared at 30 °C (*p* < 0.05) [49]. However, according to Mala et al. [43], significantly fewer *Anopheles* mosquitoes laid eggs during the dry season (38.2%) than during the wet season (61.8%) (t = 8.85, df = 1, *p* < 0.05). In addition, none of the adult mosquitoes emerged from a larval temperature of 20, 30, and 35 °C laid eggs [32].

All the studies reported on the gonotrophic cycle showed a decrease in gonotrophic cycle length with increasing temperature. The duration of the gonotrophic cycle was significantly different (X^2^ = 96.68, df = 2, *p* < 0.001) between the two seasons, as the duration of the first and second cycles was longer in the wet season (4.1 and 2.9 days, respectively) than in the dry season (3.0 and 2.2 days, respectively) [43]. In contrast, the temperature of the adult environment did not influence the probability of *An. gambiae* s.s. female mosquitoes laying eggs after their first or third blood meal. However, after the second blood meal, an increase from 23 to 31 °C, and 27 to 31 °C led to a significantly lower possibility of laying eggs (0.72 vs. 0.46, *p* = 0.002, and 0.65 vs. 0.46, *p* = 0.022, respectively) [27]. Shapiro et al. [50] also observed that the proportion of *An. stephensi* mosquitoes laying eggs was lower during the second gonotrophic cycle than the first; however, there was no noticeable effect of temperature on the probability of egg-laying in either cycle. Shapiro et al. [50] discovered that the biting rates of *An. stephensi* increased with increasing temperature. From their results, biting rates almost doubled when the temperature increased from 21 to 32 °C. The biting rate was estimated in their study as the inverse of the length of the gonotrophic cycle.

### 3.6. Effects of Temperature on the Expression of Enzymes and Susceptibility to Insecticides

Four (4) studies [15,16,29,45] assessed the effects of temperature on enzyme expression and immune responses in *Anopheles* mosquitoes (Table 6). Temperature significantly affected immune responses such as humoral melanization, defensin (DEF1), cecropin (CEC1), phagocytosis, and nitric oxide synthase (NOS) in *An. stephensi* mosquitoes. For instance, NOS expression peaked at later sampling time points in mosquitoes kept at cooler temperatures (18 °C: 24 h; 22 °C: 18 h) compared to those held at optimal or warmer temperatures (26–34 °C: 12 h) [16]. A study conducted by Murdock et al. [45] also found that NOS significantly increased at warmer temperatures (28 °C) compared to colder temperatures (20 °C vs. 28 °C, *p* = 0.002; 24 °C vs. 28 °C, *p* = 0.001). Oliver and Brooke [29] noted no significant increase in detoxification enzyme (cytochrome P450 and general esterases) systems of *An. arabiensis* mosquitoes at 25 and 37 °C.

Increasing temperature reduced the efficacy of insecticides in all three studies [18,19,29] that considered insecticide susceptibility (Table 6). Higher rearing temperatures and short-term exposure to 37 and 39 °C as adults increased pyrethroid resistance in adults of the *An. arabiensis* SENN DDT strain, and increased pyrethroid tolerance in the *An. arabiensis* SENN strain. There was a decrease in the toxicity of deltamethrin insecticide in the unselected SENN strain as the temperature increased. Likewise, increasing temperatures increased the resistance of the susceptible *An. arabiensis* strain to deltamethrin [18]. However, one study [29] observed no significant difference in mortality induced at either 37 or 39 °C for lambda-cyhalothrin (two-sample *t*-test: *p* = 0.64; t = 0.47) and permethrin (two-sample *t*-test: *p* = 0.55; t = −0.63).

## 4. Discussion

This study reviewed and assessed literature for evidence of the effects of temperature on *Anopheles* mosquito immature stages, adult life-history traits (such as fecundity, body size, length of the gonotrophic cycle, and longevity), expression of enzymes and genes, and susceptibility to insecticides. To the best of our knowledge, this is the first systematic review assessing the effects of temperature on the development of *Anopheles* mosquitoes. The mosquito’s life cycle is interdependent; thus, environmental conditions and individual characteristics in one life stage affect the other life stages [53,54,55]. An increase in temperature may have long-term repercussions on future generations [54]. The sensitivities of adult mosquitoes to temperature differ from those of the juvenile stages and life history characteristics, such as development and mortality [25].

The included studies were of good quality as most of the studies had a low risk of baseline characteristics, attrition, and reporting bias. However, some of the studies reviewed were at high risk of sequence generation and allocation concealment. This is unlikely to influence the findings. In addition, some of the included studies reared mosquitoes at a constant temperature and only exposed them to different temperature regimes prior to outcome assessment. These studies might have missed the early effects of temperature on mosquitoes; hence, the outcome of interest could be affected. The effects of rearing temperature on the immature stages can affect adult life-history traits and the overall adult fitness [21,22,56].

### 4.1. Effects of Temperature on Immature Stages of Mosquitoes

The immature stages of mosquitoes play a critical role in determining vector-borne disease dynamics. For instance, the variations in mosquito population size are determined primarily by changes that occur during larval development and growth, directly affecting the transmission of vector-borne diseases. Moreover, the larval stage’s carry-over effects can affect vectorial capacity traits such as fecundity, longevity, biting behavior, and vector competence [34].

The review revealed that an increase in temperature significantly decreased the time to pupation of *An. gambiae* s.s. larvae [34]. There is consistency in the existing literature that the rate of development of the immature stages of mosquitoes is temperature-dependent [14,57]. However, there were few inconsistencies in the effects of temperature on development times. It is unclear what could have accounted for differences in the results; further studies are needed to clarify these discrepancies. High temperatures are generally associated with faster development rates and have diverse effects on the insect’s juvenile stages [41,58]. However, extremely high (≥34 °C) temperatures delay larval development time and can induce high mortalities [14,35]. Some studies [26,27] observed that no *Anopheles* larvae survived at 35 °C. The physiological explanation underlying this is unclear; however, one of the attributable reasons is that when fourth instar larvae are developing at a faster rate, they are unable to adjust to the associated nutrient consumption, metabolism, or accumulation, which is needed for the intricate physiological process in the change from larvae to pupa [35]. In addition, thermal stress could affect the survival of immature mosquitoes [56]. The immature stages are sensitive to temperature because they usually live in small, isolated pools and cannot easily escape unfavorable environments [59]. To overcome the thermal stress experienced, mosquitoes may have to increase their metabolic rates, resulting in higher energy expenditure [60]. This could exceed oxygen supply from the environment leading to decreased performance, lowered tolerance to thermal stress [61], and the death of the mosquito.

In addition, our review showed that higher temperatures (23 to 31 °C) resulted in smaller larval sizes. This confirms the findings of Dodson et al. [61], who reported that increasing temperature resulted in smaller body sizes for *Culex tarsalis*. The mosquito’s size, especially the female, influences many epidemiologically important physiognomies, such as longevity, gonotrophic cycle length, biting rate, immunocompetence, and intensity of infection [26]. These physiognomies thus affect parasite development [62] and mosquito survival [63]. This could explain why increasing temperature significantly increased larval mortality [34]. It was noted that the way temperature affected the immature stages of mosquitoes differed from species to species, even among the same complex. However, the trend of increasing temperature with a small larval size did not change.

Only one study assessed the effects of temperature on the number of adults produced. The number of adults produced from the immature stages provides useful information in determining the population dynamics. Further studies are needed to assess how temperature influences the overall productivity (number of adults produced) of the immature stages. Furthermore, none of the studies evaluated the effects of temperature on the sex ratio of the emerged adults. The number of male and female mosquitoes emerging from the immature stages is critical in controlling mosquito populations as more males could increase the mosquito population due to increased mating probability [23].

### 4.2. Effects of Temperature on Adult Mosquitoes

#### 4.2.1. Life-History Traits

The adult mosquito’s life expectancy is sometimes shorter than the time required for the parasite to develop in the mosquito. Therefore, the longevity of the adult female mosquito is a significant factor in transmitting the parasite [25]. For example, malaria and other diseases such as dengue and filariasis require a minimum extrinsic incubation period (EIP) of 10 days before the female mosquito can be infective [64]. Before parasite transmission, the female mosquito must live longer to acquire the pathogen via a blood meal, survive beyond the extrinsic incubation period (EIP), and transmit the pathogen to a host during successive blood-feeding [64]. The review showed that increasing temperature and seasonal temperature variations decreased the longevity and increased the mortality of *Anopheles* mosquitoes. In addition, newly emerged adult mosquitoes thrived better with elevated temperatures than older mosquitoes [28]. The longevity and survival rate of *An. gambiae* showed significant seasonal variations, with much higher values observed in the rainy season (low temperature) than in the dry season (high temperature) [46]. Likewise, as temperatures increased from 15 to 35 °C, the longevity of *Anopheles* mosquitoes decreased. This is similar to other studies [65,66,67] that reported that mosquito longevity and mortality are negatively affected at higher temperatures. Increasing temperature decreased the longevity of mosquitoes and increased mosquito mortalities [66,68]. The relationship between temperature and longevity could be explained in two ways. First, higher temperatures may decrease the longevity by speeding the reaction rate of various metabolic processes that affect development and life history. Second, higher temperatures might heighten the damage caused by the by-products of metabolism, such as reactive oxygen species (ROS) [69]. This could make mosquitoes weak and induce high mortalities hence, decreasing the longevity of mosquitoes.

The review also revealed that increasing temperature reduced the body size of *Anopheles* mosquitoes. This is in agreement with the findings of Dodson et al. [61], who reported that increasing larval rearing temperature resulted in smaller body size for *Culex tarsalis*. The conditions in the larval environment can affect the size of the larvae and consequently the size of the adult mosquito [70]. Generally, mosquitoes with large body sizes have more teneral reserves carried over from the juvenile stages; hence, they live longer than those with small body sizes [34]. The size of mosquitoes affects many epidemiologically important traits, such as longevity, gonotrophic cycle length, biting rate, immunocompetence, and infection intensity [26]. Thus, these traits affected parasite development [62] and the vector’s survival [63]. Furthermore, mosquito size may affect the flight range as larger mosquitoes may have a better flight range than smaller ones [71]. In this sense, increasing temperatures may reduce the spread of mosquitoes within a locality.

It was revealed that higher temperatures decreased the fecundity of *Anopheles* mosquitoes. This corroborates data in the literature, suggesting that higher temperatures reduce mosquito fecundity [21,22,66]. However, one study [43] reported otherwise. The temperature difference between the two seasons reported in the study [43] was less than 2 °C (Table 5). Mala et al. [43] findings may not only be attributed to seasonal variation as the mosquitoes used in their study might have come from a diverse population with different genetic compositions. Furthermore, the failure of adult mosquitoes emerged from a larval temperature of 20, 30, and 35 °C to lay eggs agrees with the findings of Ezeakacha and Yee [21], who recorded no eggs laid by *Aedes albopictus* at the adult temperature of 20 °C in all the larval rearing temperatures used. The inability of mosquitoes to lay eggs at these temperatures could be that females were unmated, therefore, unable to produce mature eggs [21]. These studies did not check the spermathecae of females or mating status of mosquitoes. It is possible that mosquito mating may be affected by temperature. It would be of great interest for future studies to explore the effects of temperature on the mating success of *Anopheles* mosquitoes. This could provide useful information in controlling *Anopheles* mosquitoes in a future warmer climate.

Usually, higher temperatures may accelerate the digestion of blood meals, reduce the gonotrophic cycle’s length, and modify mosquito fecundity [72]. Our review supports this as increasing temperature reduced the length of the gonotrophic cycle of *Anopheles* mosquitoes. An increase in temperature could fast-track blood meal digestion and lessen the gonotrophic cycle length [43]. Lardeux et al. [73] observed that an increase in temperature from 15 to 31 °C drastically reduced the length of the gonotrophic cycle of *An. pseudopunctipennis* from approximately nine to two days. Naturally, a relatively small number of female mosquitoes survive for quite a long period to complete more than two gonotrophic cycles [74]. Therefore, any decrease in the gonotrophic cycle length can boost malaria incidence due to the increased frequency of egg-laying and biting rates of mosquitoes [43].

Only one study reported the relationship between temperature and biting rate [50]. They observed that increasing the temperature from 21 to 32 °C increased the biting rates of *An. stephensi* mosquitoes. This may be attributed to the effects of temperature on a blood meal. Increasing temperature speeds blood meal digestion, leading to increased host biting rates [14]. The female mosquito bites its host to acquire a blood meal, which is needed to develop its eggs [75]. Blood feeding and egg production are closely related, and blood-feeding is crucial for the female mosquito to acquire the malaria parasite and transfer it to its host [76]. Thus, any factor that affects the biting rate has a detrimental effect on mosquito’s ability to produce eggs and transmit diseases. An increase in mosquito biting rate implies that the vector may feed more frequently on its host and increase its potential to transmit diseases [14].

#### 4.2.2. Expression of Enzymes, Immune Responses, and Susceptibility to Insecticides

High temperatures modify biochemical processes, increase metabolic rates [29], and affect the mosquito’s immune system [15,16,17]. It has been shown that temperature can have a striking and diverse qualitative and quantitative effect on mosquito’s immune responses by affecting the immune challenge time and nature [16]. The review on the expression of immune responses suggested that there were complex interactions between time, temperature, and the type of immune challenge. Most of the immune responses studied by Murdock et al. [16] were more robust at low temperature (18 °C) than high temperature. This is consistent with the findings of Suwanchaichinda and Paskewitz [77], who reported that the percentage of female *An. gambiae* heavily melanizing beads were highest when held at 24 °C compared to 27 and 30 °C. In addition to innate immunity, melanin production plays a crucial role in physiological processes such as cuticular tanning and egg hardening, explaining the fast rate of Humoral Melanization at lower or cooler temperatures [16]. In addition, NOS expression significantly increased at warmer temperatures (i.e., 28 °C) relative to colder temperatures [45], which is consistent with similar studies [15,16]. According to Shapiro et al. [50], their model suggested 29 °C as the optimum temperature required for malaria transmission. Therefore, an increase in NOS expression at higher temperatures could be an essential mosquito defense that could hinder parasite development [16].

Only one of the studies reviewed [29] assessed the effects of temperature on detoxification enzyme activity (cytochrome P450 and general esterases). It showed that the detoxification enzyme systems of the mosquitoes were not significantly affected by an increase in temperature. It is unclear what could have accounted for the lack of significant effect of temperature on detoxification enzyme expression. Further studies are needed to investigate the effects of rearing temperatures on the expression of detoxification enzymes in *Anopheles* mosquitoes. Temperature affects mosquito nervous system sensitivity, immune responses, and metabolic activities, consequently influencing the efficacy of insecticides [78]. None of the studies considered the effects of temperature on target site resistance–one of the most common and well-studied forms of insecticide resistance [79,80,81,82]. Generally, metabolic and target site resistance can co-occur in the same population [83] and can lead to complex cross-resistance and high resistance levels [84]. It is unclear how higher or warmer temperatures will shift metabolic rates and target site insensitivity in mosquitoes, especially *Anopheles* species.

For susceptibility, it was revealed that higher temperatures reduced insecticide toxicity in *An. funestus* and *An. arabiensis* mosquitoes. The reduced toxicity at high temperatures might be due to higher enzymatic activities, which could increase detoxification of the insecticide [85]. In addition, how temperature affected the toxicity of deltamethrin differed from that of bendiocarb. However, the synergistic PBO completely restored pyrethroid susceptibility irrespective of the temperature. The difference in the toxicity of the two insecticides could be attributed to the differences in the mode of action. Bendiocarb, which belongs to carbamates, are nerve poisons that work by inhibiting acetylcholinesterase. On the other hand, deltamethrin belonging to pyrethroids alters the normal function of insect nerves by modifying the kinetics of voltage-sensitive sodium channels [86].

This review further revealed that the mosquito strain played a critical role in how temperature affected the toxicity of deltamethrin, and its temperature coefficient was not always positive or negative [18]. This is consistent with the findings of Hodjati and Curtis [87], who also found that the toxicity of 0.25% permethrin on resistant *An. stephensi* exhibited a slight negative temperature coefficient (between 16 °C and 28 °C) and a strongly positive temperature coefficient (between 28 °C and 37 °C). Many mechanisms have been ascribed to the reduced efficacy of insecticides at elevated temperatures. For instance, pyrethroid insecticides are axonic poisons and control sodium ions’ movement during nerve impulse movement. Generally, neuron sensitivity declines between temperatures of 30 to 35 °C, which influences the efficacy of insecticides. In addition, at low temperatures, neurons exposed to pyrethroid insecticides receive a high concentration of the insecticide due to reduced biotransformation. This makes the neuron more sensitive to the resulting stimulus because of a prolonged duration of steady-state resting potential [88].

It needs to be emphasized that mosquito rearing temperature is critical, as it may influence the quality of the adult mosquito [24] and its susceptibility to insecticides. The rearing, exposure, and postexposure temperatures can influence mosquito susceptibility to insecticides [19]. Besides, the association between temperature and insecticide efficacy differs based on the mode of action of an insecticide, method of application, target species, and quantity of insecticide contacted or ingested by the target species [89].

### 4.3. Implications of Findings for Malaria Control in a Future Warmer Climate

Climate change is anticipated to shift the distribution of vector-borne diseases such as malaria [90]. Both the malaria vector and the parasite itself are sensitive to climate parameters, particularly temperature and rainfall [90]. Studies have reported that variations in climate parameters profoundly affect the development of malaria parasites and mosquito longevity, which ultimately affects malaria transmission [91].

Both extreme low and high temperatures affect mosquito development and survival [42]. Studies have reported the effects of extreme low and high temperatures on the development of the malaria parasite. For instance, Mordecai et al. [92] indicated that both insect and parasite physiology limit malaria transmission to temperatures between 17 and 34 °C. At a temperature of 25 °C, the malaria parasite needs only 12 days to complete its development; however, over 30 days is required for the parasite to develop and become infectious when the temperature is 20 °C [93]. This is very important for malaria control because if parasite development takes a longer time, then the likelihood that a mosquito will survive longer for the parasite to transmit the disease will decrease drastically [94]. On the other hand, the development of *An. gambiae* is greatly impeded when temperatures are low, and its larvae are unable to develop and die at temperatures below 16 and 14 °C, respectively [14].

The fate of malaria control in a future warmer climate can be seen from two directions. First, in a future warmer climate, areas that are currently cold (below 17 °C) and do not support the survival of malaria vectors and parasites to complete their development could provide suitable conditions for their survival and development due to an increase in temperature. The second direction that may be considered as the great news is that if the mosquitoes and the parasite fail to adapt to increasing temperatures, especially in currently warmer areas (temperatures above 34 °C), such as sub-Saharan Africa, then these areas could start experiencing a reduction in malaria cases. Ultimately, these countries can eradicate the disease because mosquitoes may not survive long to complete the parasite incubation period at temperatures higher than 34 °C [26,35]. It is noteworthy that factors such as plasticity, adaptation, thermal regulation, and daily, monthly, and seasonal climatic variations, and microclimates [48,95] may influence malaria transmission. However, these factors were not included in this review.

## 5. Conclusions

This review had some limitations. The search strategy used might not have captured all studies related to the topic. However, by searching a wide range of databases and a reference list of articles, we believe that all major studies on *Anopheles* mosquitoes and temperature might have been captured. Besides, we only included articles written in the English language; nonetheless, we believe it is unlikely to have resulted in the omission of any major paper in the area. Another limitation has to do with the rearing of mosquitoes. In some of the included studies, adult mosquitoes were only exposed to the selected temperature regimes only before outcome assessment, which may not accurately estimate the effects of temperature on the outcome. To measure the impact of temperature, future studies should consider rearing mosquitoes in the selected temperature regimes at the egg stage through to the stage required for outcome assessment.

Despite the limitations stated, this review revealed that *Anopheles* mosquitoes are susceptible to mean environmental temperature and temporal variations. Many life-history traits of *Anopheles* mosquitoes, such as longevity, biting rate, fecundity, body size, length of the gonotrophic cycle, adult and larval development, and expression of enzymes and susceptibility to insecticides, are greatly affected by temperature. This suggests that higher temperatures expected in a warmer climate could have diverse effects on *Anopheles* mosquitoes. This may affect the population dynamics and ecology and the disease transmission potential of these mosquitoes.

Though most of the included studies were of similar design (laboratory- and field-based studies), there was some variation in the methods or techniques used in rearing the mosquitoes. Few studies considered the effects of temperature on the length of the gonotrophic cycle, biting rate, fecundity, and enzyme expression. The sensitivities of *Anopheles* mosquitoes to temperature differ from species to species, even among the same complex. Notwithstanding, there seem to be limited studies on the effects of temperature on adult life-history traits of *Anopheles* mosquitoes, and more studies are needed to clarify this relationship. To forecast malaria transmission and the effectiveness of control measures in a future warmer climate, a deeper understanding of this complexity and its mechanisms are required to understand and model the effects of temperature on the immature stages, life-history traits, insecticide susceptibility, and expression of enzymes in the adult *Anopheles* mosquitoes.

## Figures and Tables

**Table 1:** Risk of bias in included studies using the SYRCLE tool.

Author/Year	Sequence Generation (Selection Bias)	Baseline Characteristics (Selection Bias)	Allocation Concealment (Selection Bias)	Incomplete Outcome Data (Attrition Bias)	Selective Reporting (Reporting Bias)	Other Bias (Rearing of Mosquito)	Other Bias (Funding Source)
Aytekin et al. [32]	High risk	Low risk	Unclear risk	Low risk	Low risk	Low risk	High risk
Barreaux et al. [33]	High risk	Low risk	Unclear risk	Low risk	Low risk	Low risk	High risk
Barreaux et al. [34]	High risk	Low risk	Unclear risk	Low risk	Low risk	Low risk	Low risk
Bayoh and Lindsay [35]	High risk	Low risk	Unclear risk	Low risk	Low risk	Low risk	Low risk
Bayoh and Lindsay [36]	High risk	Low risk	Unclear risk	Low risk	Low risk	Low risk	Low risk
Charlwood and Bragança [37]	High risk	Unclear risk	Unclear risk	Low risk	Low risk	High risk	High risk
Christiansen-Jucht et al. [26]	High risk	Low risk	Unclear risk	Low risk	Low risk	Low risk	Low risk
Christiansen-Jucht et al. [27]	High risk	Low risk	Unclear risk	Low risk	Low risk	Low risk	Low risk
Davies et al. [38]	High risk	Low risk	Unclear risk	Low risk	Low risk	Low risk	Low risk
Faiman et al. [39]	High risk	Low risk	Unclear risk	Low risk	Low risk	Unclear risk	Low risk
Glunt et al. [18]	High risk	Low risk	High risk	Low risk	Low risk	High risk	Low risk
Glunt et al. [19]	High risk	Low risk	High risk	Low risk	Low risk	High risk	Low risk
Impoinvil et al. [40]	High risk	Low risk	Unclear risk	Low risk	Low risk	Low risk	Low risk
Kirby and Lindsay [41]	High risk	Low risk	Unclear risk	Low risk	Low risk	Low risk	High risk
Lyons et al. [42]	High risk	Low risk	High risk	Low risk	Low risk	Low risk	Low risk
Lyons et al. [28]	High risk	Low risk	High risk	Low risk	Low risk	High risk	Low risk
Mala et al. [43]	Low risk	Low risk	High risk	Low risk	Low risk	High risk	Low risk
Mamai et al. [44]	High risk	Low risk	High risk	Low risk	Low risk	Low risk	Low risk
Murdock et al. [45]	High risk	Low risk	High risk	Low risk	Low risk	High risk	Low risk
Murdock et al. [15]	High risk	Low risk	High risk	Low risk	Low risk	High risk	Low risk
Murdock et al. [16]	High risk	Low risk	High risk	Low risk	Low risk	High risk	Low risk
Olayemi et al. [46]	High risk	Unclear risk	High risk	Low risk	Low risk	High risk	High risk
Oliver and Brooke [29]	High risk	Low risk	High risk	Low risk	Low risk	Low risk	Low risk
Paaijmans et al. [47]	High risk	Low risk	High risk	Low risk	Low risk	Low risk	High risk
Paaijmans et al. [48]	High risk	Low risk	High risk	Low risk	Low risk	Low risk	Low risk
Phasomkusolsil et al. [49]	High risk	Low risk	High risk	Low risk	Low risk	Low risk	High risk
Rúa et al. [51]	High risk	Low risk	High risk	Low risk	Low risk	Low risk	High risk
Shapiro et al. [50]	High risk	Low risk	High risk	Low risk	Low risk	Low risk	Unclear risk
Wallace and Merritt [52]	High risk	Low risk	High risk	Low risk	Low risk	Low risk	Low risk

NB: Performance (Random housing and Blinding) and Detection (Random outcome assessment and Blinding) biases were not applicable.

**Table 2:** Effects of temperature on immatures stages of *Anopheles* mosquitoes.

Author, Year	Study Type	Study Location	Species Considered	Conditions	Outcome Considered
Christiansen-Jucht et al. [27]	Laboratory-based	United Kingdom	*An. gambiae* s.s.	23, 27, 31, and 35 ± 1 °C 12:12 (L:D) photoperiod RH 75% ± 5%	Egg hatching time ** Development time * Larval size **
Davies et al. [38]	Laboratory-based	South Africa	*An. arabiensis* *An. quadriannulatus*	25, 20–30, and 18–35 °C 12:12 (L:D) photoperiod RH 80%	Egg hatching time ** Development time ** Larval survival **
Impoinvil et al. [40]	Laboratory-based	Kenya	*An. gambiae* s.s.	Immature: 30–35 °C Adult: 22–27 °C RH 80–90%	Egg hatching count *
Mamai et al. [44]	Laboratory-based	Austria	*An. arabiensis*	22 ± 1 °C, 22–27 ± 1 °C, 27 ± 1 °C 12:12 (L:D) photoperiod RH 80%	Egg hatching time ** Pupation success
Phasomkusolsil et al. [49]	Laboratory-based	Thailand	*An. dirus* *An. sawadwongporni*	23 and 30 °C	Egg hatching time Development time **
Aytekin et al. [32]	Laboratory-based	Turkey	*An. superpictus*	15, 20, 25, 27, 30, and 35 °C, 12:12 (L:D) photoperiod RH 65% ± 5%	Egg hatching count Development time ** Larval survival
Bayoh and Lindsay [35]	Laboratory-based	United Kingdom	*An. gambiae* s.s.	10 to 40 °C (±1 °C), with 2 °C increments 12:12 (L:D) photoperiod RH 80% ± 10%	Development time ** Adult emergence **
Kirby and Lindsay [41]	Laboratory-based	United Kingdom	*An. gambiae* s.s. *An. arabiensis*	25, 30, or 35 ◦C	Development time ** Larval survival **
Lyons et al. [42]	Laboratory-based	South Africa	*An. arabiensis* *An. funestus*	15, 18, 20, 22, 25, 28, 30, 32 35, 15 °C–35, and 20–30 °C 12:12 (L:D) photoperiod RH 80%	Development time ** Survival of immature stages
Oliver and Brooke [29]	Laboratory-based	South Africa	*An. arabiensis*	25, 30, and 35 °C RH 80% ± 5%	Development time **
Paaijmans et al. [48]	Laboratory-based	United States of America	*An. stephensi*	16 to 36 °C, with 2 °C increments	Development time ** Larval survival **
Wallace and Merritt [52]	Field and Laboratory-based	United States of America	*An. quadrimaculatus*	18, 23, and 28 °C	Larval survival **
Lyons et al. [28]	Laboratory-based	South Africa	*An. funestus* *An. arabiensis*	20, 25, and 30 °C 12:12 (L:D) photoperiod RH 80%	Larval survival **
Bayoh and Lindsay [36]	Laboratory-based	United Kingdom	*An. gambiae* s.s.	10 to 40 °C (±1 °C), with 2 °C increments 12:12 (L:D) photoperiod RH 80 ± 10%	Larval survival **
Christiansen-Jucht et al. [26]	Laboratory-based	United Kingdom	*An. gambiae* s.s.	23, 27, 31, and 35 ± 1 °C 12:12 (L:D) photoperiod RH 75% ± 5%	Larval survival **
Barreaux et al. [34]	Laboratory-based	Switzerland	*An. gambiae* s.s.	21, 25, and 29 °C 12:12 (L:D) photoperiod RH 70% ± 5%	Time to pupation **

Outcomes with a single asterisk (*) indicate that higher temperatures generally increased the outcomes; Outcomes with a double asterisk (**) indicate that higher temperatures generally decreased those outcomes; Outcomes with no asterisk indicate no significant effect of temperature.

**Table 3:** Effects of temperature on the longevity of *Anopheles* mosquitoes.

Author, Year	Study Type	Study Location	Species Considered	Conditions	Outcome Considered
Aytekin et al. [32]	Laboratory-based	Turkey	*An. superpictus*	15, 20, 25, 27, 30, and 35 °C, 12:12 (L:D) photoperiod RH 65% ± 5%	Longevity **
Barreaux et al. [34]	Laboratory-based	Switzerland	*An. gambiae* s.s.	21, 25, and 29 °C 12:12 (L:D) photoperiod RH 70% ± 5%	Longevity
Faiman et al. [39]	Laboratory-based	United States of America	*An. coluzzii*	22, 23.5, and 27 °C, 2:12 or 11:13 L:D photoperiod RH 85% and 50%	Longevity **
Olayemi et al. [46]	Field and Laboratory-based	Nigeria	*An. gambiae*	Seasons Dry: 31.12 ± 1.09 °C, RH 44.01 ± 7.02% Rainy: 27.67 ± 1.27 °C, RH 69.51% ± 12.44%	Longevity **
Oliver and Brooke [29]	Laboratory-based	South Africa	*An. arabiensis*	25, 30, and 35 °C RH 80% ± 5%	Longevity **

Outcomes with a single asterisk (*) indicate that higher temperatures generally increased the outcomes; Outcomes with a double asterisk (**) indicate that higher temperatures generally decreased those outcomes; Outcomes with no asterisk indicate no significant effect of temperature.

**Table 4:** Effects of temperature on the body size of *Anopheles* mosquitoes.

Author, Year	Study Type	Study Location	Species Considered	Conditions	Outcome Considered
Aytekin et al. [32]	Laboratory-based	Turkey	*An. superpictus*	15, 20, 25, 27, 30, and 35 °C, 12:12 (L:D) photoperiod RH 65% ± 5%	Body size **
Barreaux et al. [33]	Laboratory-based	Switzerland	*An. gambiae* s.s.	21 °C, 25 °C, and 29 °C	Body size **
Barreaux et al. [34]	Laboratory-based	Switzerland	*An. gambiae* s.s.	21, 25, and 29 °C 12:12 (L:D) photoperiod RH 70% ± 5%	Body size **
Charlwood and Bragança [37]	Field-based	Mozambique	*An. funestus*	17 to 33 °C	Body size **
Christiansen-Jucht et al. [27]	Laboratory-based	United Kingdom	*An. gambiae* s.s.	23, 27, 31, and 35 ± 1°C 12:12 (L:D) photoperiod RH 75% ± 5%	Body size **
Kirby and Lindsay [41]	Laboratory-based	United Kingdom	*An. gambiae* s.s. *An. arabiensis*	25, 30 or 35 °C	Body size **
Phasomkusolsil et al. [49]	Laboratory-based	Thailand	*An. dirus* *An. sawadwongporni*	23 and 30 °C	Body size **

The double asterisk (**) indicates that higher temperatures generally decreased those outcomes.

**Table 5:** Effects of temperature on fecundity, length of the gonotrophic cycle, and biting rate of *Anopheles* mosquitoes.

Author, Year	Study Type	Study Location	Species Considered	Conditions	Outcome Considered
Aytekin et al. [32]	Laboratory-based	Turkey	*An. superpictus*	15, 20, 25, 27, 30, and 35 °C, 12:12 (L:D) photoperiod RH 65% ± 5%	Fecundity **
Christiansen-Jucht et al. [27]	Laboratory-based	United Kingdom	*An. gambiae* s.s.	23, 27, 31, and 35 ± 1 °C 12:12 (L:D) photoperiod RH 75% ± 5%	Fecundity **
Phasomkusolsil et al. [49]	Laboratory-based	Thailand	*An. dirus* *An. sawadwongporni*	23 and 30 °C	Fecundity **
Mala et al. [43]	Field-based	Kenya	*An. arabiensis* *An. pharaoensis* *An. coustani* *An. funestus*	Indoor Temperature Dry season (28.22 ± 1.1 °C) Rainy season (27.12 ± 1.2 °C) Outdoor Temperature Dry season (26.32 ± 0.33 °C) Rainy season (24.82 ± 0.33 °C)	Fecundity * Gonotrophic cycle **
Paaijmans et al. [47]	Laboratory-based	United States of America	*An. stephensi*	22, 24, and 26 °C 12:12 (L:D) photoperiod RH 90% ± 5%	Gonotrophic cycle **
Rúa et al. [51]	Laboratory-based		*An. albimanus*	24, 27, and 30 °C	Gonotrophic cycle **
Shapiro et al. [50]	Laboratory-based	United States of America	*An. stephensi*	21, 24, 27, 30, 32, and 34 °C	Gonotrophic cycle ** Biting rate *

Single asterisk (*) indicates that higher temperatures generally increased the outcomes, Double asterisk (**) indicates that higher temperatures generally decreased those outcomes.

**Table 6:** Effects of temperature on insecticide susceptibility, expression of enzymes and immune responses in *Anopheles* mosquitoes.

Author, Year	Study Type	Study Location	Species Considered	Conditions	Outcome Considered
Glunt et al. [18]	Laboratory-based	South Africa	*An. funestus* *An. arabiensis*	18 °C, 25 °C, and 30 °C RH 70% for 18 °C and 30 °C RH 80% for 25 °C	Insecticide susceptibility (deltamethrin, bendiocarb, synergist PBO) **
Glunt et al. [19]	Laboratory-based	United States of America	*An. stephensi*	12, 18, 22, and 26 °C	Insecticide susceptibility (malathion, permethrin)
Oliver and Brooke [29]	Laboratory-based	South Africa	*An. arabiensis*	25, 30, and 35 °C RH 80% ± 5%	Insecticide susceptibility Detoxification enzyme activity
Murdock et al. [45]	Laboratory-based	United States of America	*An. stephensi*	20, 22, 24, 26, and 28 ± 0.5 °C 12:12 (L:D) photoperiod RH 80% ± 5%	Nitric oxide synthase *
Murdock et al. [15]	Laboratory-based	United States of America	*An. stephensi*	16, 26, 32 ± 0.5 °C; 16, 26, 32 ± 6 °C 12:12 (L:D) photoperiod RH 80% ± 5%	Defensin Cecropin Nitric oxide synthase
Murdock et al. [16]	Laboratory-based	United States of America	*An. stephensi*	12, 18, 24, 28, and 34 + 0.5 °C 12:12 (L:D) photoperiod RH 80% ± 5%	Humoral Melanization Cecropin Phagocytosis** Defensin Nitric oxide synthase *

Outcomes with a single asterisk (*) indicate that higher temperatures generally increased the outcomes; Outcomes with a double asterisk (**) indicate that higher temperatures generally decreased those outcomes; Outcomes with no asterisk indicate no significant effect of temperature.

## Data Availability

The datasets supporting the conclusions of this article are included within the manuscript and its Supplementary Materials.

## Supplementary Materials

The following are available online at https://www.mdpi.com/article/10.3390/ijerph18147255/s1, Table S1: Search terms and search results from databases; Table S2: List of studies excluded with reasons.

## Abbreviations

DDT	Dichlorodiphenyltrichloroethane
EIP	Extrinsic incubation period
NOS	Nitric oxide synthase
PBO	Piperonyl butoxide
PRISMA	Preferred reporting items for systematic reviews and meta-analyses
ROS	Reactive oxygen species
SYRCLE	Systematic review center for laboratory animal experimentation
WHO	World health organization
