# Supplementary material for: A Systematic Review of the Effects of Temperature on Anopheles Mosquito Development and Survival: Implications for Malaria Control in a Future Warmer Climate
Source: Int J Environ Res Public Health. 2021 Jul 7;18(14):7255. doi: 10.3390/ijerph18147255 (PMC8306597; doi:10.3390/ijerph18147255)
Supplement: Supplementary file 1 [file ijerph-18-07255-s001.zip › ijerph-1226650-supplementary.pdf]

Supplementary information

Table S1: Search terms and search results from databases

Database	Search Term	Search Results
Google Scholar	("Anopheles mosquito") AND ("temperature" OR "season") AND ("longevity" OR "fecundity" OR "gonotrophic cycle" OR "development time" OR "enzyme" OR "insecticide")	**8130**
Scopus	TITLE-ABS-KEY ("anopheles mosquito" OR "malaria") AND ("temperature" OR season) AND ("development time" OR "survival" OR "longevity" OR "fecundity" OR "gonotrophic cycle" OR "biting rate" OR "enzyme" OR "insecticide")	**5926**
Science Direct	("Anopheles mosquito") AND ("temperature" OR "season") AND ("longevity" OR "fecundity" OR "gonotrophic cycle" OR "development time" OR "enzyme" OR "insecticide")	**1403**
PubMed	("anopheles mosquito" OR "malaria") AND ("temperature" OR season) AND ("development time" OR "survival" OR "longevity" OR "fecundity" OR "gonotrophic cycle" OR "biting rate" OR "enzyme" OR "insecticide")	**1156**
ProQuest	("Anopheles mosquito") AND ("temperature" OR "season") AND ("longevity" OR "fecundity" OR "gonotrophic cycle" OR "development time" OR "enzyme" OR "insecticide")	**850**
Web of Science	("Anopheles mosquito") AND ("temperature" OR "season") AND ("longevity" OR "fecundity" OR "gonotrophic cycle" OR "development time" OR "enzyme" OR "insecticide")	**17**
Hand search		4

Table S2: List of studies excluded with reasons

Sn	Author name	Reason for exclusion
1	Abiodun et al. [1]	1
2	Abiodun et al. [2]	1
3	Abiodun et al. [3]	1
4	Agusto et al. [4]	1
5	Beck-Johnson [5]	1
6	Beck-Johnson et al. [6]	1
7	Beck-Johnson et al. [7]	1
8	Blanford et al. [8]	2
9	Charlwood and Braganca [9]	3
10	Christiansen-Jucht et al. [10]	1
11	Culler et al. [11]	1
12	Ewing et al. [12]	1
13	Glunt et al. [13]	2
14	Gonçalvez De Carvalho et al. [14]	3
15	Lindsay et al. [15]	4
16	Lunde et al. [16]	1
17	Lyons et al. [17]	1
18	Mordecai et al. [18]	2
19	Mukhtar et al. [19]	1
20	Murdock et al. [20]	2
21	Murdock et al. [21]	2
22	Okechukwu et al. [22]	4
23	Okuneye et al. [23]	1
24	Owiti and Christopher [24]	3
25	Owusu et al. [25]	2
26	Paaijmans et al. [26]	3
27	Paaijmans et al. [27]	2
28	Paaijmans et al. [28]	1
29	Parham and Michael [29]	1
30	Parham and Michael [30]	1
31	Parham et al. [31]	1
32	Shimaponda-Mataa et al. [32]	1
33	Wang et al. [33]	1
34	Wei-Ming et al. [34]	4
35	Yamana and Eltahir [35]	1
36	Zakharova et al. [36]	4

1 = mathematical models; 2 = Opinions/Letters/Reports; 3 = Study did not report on any of the outcomes of interest; 4 = Full text not available

References

1. Abiodun, G.J., Maharaj, R., Witbooi, P., and Okosun, K.O., *Modelling the influence of temperature and rainfall on the population dynamics of Anopheles arabiensis.* Malaria Journal, 2016. **15**(1): p. 364.
2. Abiodun, G.J., Witbooi, P., and Okosun, K.O., *Modeling and analyzing the impact of temperature and rainfall on mosquito population dynamics over Kwazulu-Natal, South Africa.* International Journal of Biomathematics, 2017. **10**(4).
3. Abiodun, G.J., Witbooi, P., and Okosun, K.O., *Modelling the impact of climatic variables on malaria transmission.* Hacettepe Journal of Mathematics and Statistics, 2018. **47**(2): p. 219-235.

4. Agusto, F., Gumel, A., and Parham, P., *Qualitative assessment of the role of temperature variations on malaria transmission dynamics.* Journal of Biological Systems, 2015. **23**(04): p. 1550030.
5. Beck-Johnson, L.M., *Temperature impacts on mosquito population dynamics and malaria transmission.* 2013.
6. Beck-Johnson, L.M., Nelson, W.A., Paaijmans, K.P., Read, A.F., Thomas, M.B., and Bjørnstad, O.N., *The effect of temperature on Anopheles mosquito population dynamics and the potential for malaria transmission.* PLOS ONE, 2013. **8**(11): p. e79276.
7. Beck-Johnson, L.M., Nelson, W.A., Paaijmans, K.P., Read, A., Thomas, M.B., and Bjørnstad, O.N., *The importance of temperature fluctuations in understanding mosquito population dynamics and malaria risk.* Royal Society Open Science, 2017. **4**(3).
8. Blanford, J.I., Blanford, S., Crane, R.G., Mann, M.E., Paaijmans, K.P., Schreiber, K.V., et al., *Implications of temperature variation for malaria parasite development across Africa.* Scientific reports, 2013. **3**(1): p. 1-11.
9. Charlwood, J.D. and Braganca, M., *The effect of rainstorms on adult Anopheles funestus behavior and survival.* Journal of Vector Ecology, 2012. **37**(1): p. 252-256.
10. Christiansen-Jucht, C., Erguler, K., Shek, C.Y., Basanez, M.-G., and Parham, P.E., *Modelling Anopheles gambiae ss population dynamics with temperature-and age-dependent survival.* International journal of environmental research public health, 2015b. **12**(6): p. 5975-6005.
11. Culler, L.E., Ayres, M.P., and Virginia, R.A., *In a warmer Arctic, mosquitoes avoid increased mortality from predators by growing faster.* Proceedings of the Royal Society B: Biological Sciences, 2015. **282**(1815): p. 20151549.
12. Ewing, D.A., Cobbold, C.A., Purse, B., Nunn, M., and White, S.M., *Modelling the effect of temperature on the seasonal population dynamics of temperate mosquitoes.* Journal of theoretical biology, 2016. **400**: p. 65-79.
13. Glunt, K.D., Blanford, J.I., and Paaijmans, K.P., *Chemicals, climate, and control: increasing the effectiveness of malaria vector control tools by considering relevant temperatures.* PLoS Pathogens, 2013. **9**(10): p. e1003602.
14. Gonçalvez De Carvalho, S.C., De Jesus Martins Jr, A., Pereira Lima, J.B., and Valle, D., *Temperature influence on embryonic development of Anopheles albitarsis and Anopheles aquasalis.* Memorias do Instituto Oswaldo Cruz, 2002. **97**(8): p. 1117-1120.
15. Lindsay, S.W., Wilkins, H.A., Zieler, H.A., Daly, R.J., Petrarca, V., and Byass, P., *Ability of Anopheles gambiae mosquitoes to transmit malaria during the dry and wet seasons in an area of irrigated rice cultivation in The Gambia.* Journal of Tropical Medicine and Hygiene, 1991. **94**(5): p. 313-324.
16. Lunde, T.M., Bayoh, M.N., and Lindtjørn, B., *How malaria models relate temperature to malaria transmission.* Parasites and Vectors, 2013. **6**(1): p. 1-10.
17. Lyons, C.L., Coetzee, M., Terblanche, J.S., and Chown, S.L., *Desiccation tolerance as a function of age, sex, humidity and temperature in adults of the African malaria vectors Anopheles arabiensis and Anopheles funestus.* Journal of Experimental Biology, 2014. **217**(21): p. 3823-3833.
18. Mordecai, E.A., Paaijmans, K.P., Johnson, L.R., Balzer, C., Ben-Horin, T., de Moor, E., et al., *Optimal temperature for malaria transmission is dramatically lower than previously predicted.* Ecology letters, 2013. **16**(1): p. 22-30.
19. Mukhtar, A.Y.A., Munyakazi, J.B., and Ouifki, R., *Assessing the role of climate factors on malaria transmission dynamics in South Sudan.* Mathematical Biosciences, 2019. **310**: p. 13-23.
20. Murdock, C., Sternberg, E., and Thomas, M., *Malaria transmission potential could be reduced with current and future climate change.* Scientific reports, 2016. **6**(1): p. 1-7.
21. Murdock, C.C., Paaijmans, K.P., Cox-Foster, D., Read, A.F., and Thomas, M.B., *Rethinking vector immunology: the role of environmental temperature in shaping resistance.* Nature Reviews Microbiology, 2012. **10**(12): p. 869.
22. Okechukwu, R., Okereke, J., Ezejiofor, T., Obasi, K., and Ebere, G., *Relationship between malaria vector densities in artificial container habitats, land-use changes and temperature.* Nigerian Journal of Parasitology, 2011. **32**(2).

23. Okuneye, K., Eikenberry, S.E., and Gumel, A.B., *Weather-driven malaria transmission model with gonotrophic and sporogonic cycles.* Journal of biological dynamics, 2019. **13**(sup1): p. 288-324.
24. Owiti, Y.J. and Christopher, M.J.J.J.o.B.S., *Effect of Temperature and pH on Egg Viability and Pupation of Anopheles arabiensis Patton (Diptera: Culicidae): Prospect for Optimizing Colony Reproduction Procedures.* 2017. **10**(1).
25. Owusu, H.F., Chitnis, N., and Müller, P., *Insecticide susceptibility of Anopheles mosquitoes changes in response to variations in the larval environment.* Scientific reports, 2017. **7**(1): p. 1-9.
26. Paaijmans, K.P., Blanford, S., Chan, B.H.K., and Thomas, M.B., *Warmer temperatures reduce the vectorial capacity of malaria mosquitoes.* Biology Letters, 2012. **8**(3): p. 465-468.
27. Paaijmans, K.P., Imbahale, S.S., Thomas, M.B., and Takken, W., *Relevant microclimate for determining the development rate of malaria mosquitoes and possible implications of climate change.* Malaria Journal, 2010. **9**(1).
28. Paaijmans, K.P., Read, A.F., and Thomas, M.B., *Understanding the link between malaria risk and climate.* Proceedings of the National Academy of Sciences of the United States of America, 2009. **106**(33): p. 13844-13849.
29. Parham, P.E. and Michael, E., *Modelling climate change and malaria transmission*, in *Advances in Experimental Medicine and Biology*. 2010a. p. 184-199.
30. Parham, P.E. and Michael, E., *Modeling the effects of weather and climate change on malaria transmission.* Environmental health perspectives, 2010b. **118**(5): p. 620-626.
31. Parham, P.E., Pople, D., Christiansen-Jucht, C., Lindsay, S., Hinsley, W., and Michael, E., *Modeling the role of environmental variables on the population dynamics of the malaria vector Anopheles gambiae sensu stricto.* Malaria Journal, 2012. **11**.
32. Shimaponda-Mataa, N.M., Tembo-Mwase, E., Gebreslasie, M., Achia, T.N., and Mukaratirwa, S., *Modelling the influence of temperature and rainfall on malaria incidence in four endemic provinces of Zambia using semiparametric Poisson regression.* Acta tropica, 2017. **166**: p. 81-91.
33. Wang, X., Tang, S., and Cheke, R.A., *A stage structured mosquito model incorporating effects of precipitation and daily temperature fluctuations.* Journal of theoretical biology, 2016. **411**: p. 27-36.
34. Wei-Ming, W., Hua-Yun, Z., Jun, C., Guo-Jing, Y., Ju-Lin, L., Ya-Ping, G., et al., *Impact of different temperatures on development of Anopheles anthropoghagus in Jiangsu Province.* Zhongguo xue xi chong bing fang zhi za zhi = Chinese journal of schistosomiasis control, 2011. **23**(5): p. 571-574.
35. Yamana, T.K. and Eltahir, E.A., *Incorporating the effects of humidity in a mechanistic model of Anopheles gambiae mosquito population dynamics in the Sahel region of Africa.* Parasites and vectors, 2013. **6**(1): p. 1-10.
36. Zakharova, N.F., Losev, G.I., and Iakubovich, V., *The effect of density and temperature on the larval populations of the malaria vector Anopheles sacharovi.* Med Parazitol (Mosk), 1990(1): p. 3-7.
